# Acupuncture as a therapeutic treatment option for threatened miscarriage

**DOI:** 10.1186/1472-6882-12-20

**Published:** 2012-03-22

**Authors:** Debra Betts, Caroline A Smith, Dahlen G Hannah

**Affiliations:** 1 Centre for Complementary Medicine Research, University of Western Sydney, Penrith South, NSW 2751, Australia; 2 School of Nursing and Midwifery, University of Western Sydney, Penrith South, NSW 2751, Australia

## Abstract

**Background:**

Threatened miscarriage involves vaginal bleeding in a pregnancy that remains viable. This is a common early pregnancy complication with increased risk factors for early pregnancy loss, preterm premature rupture of membranes (PPROM), preterm delivery, low birth weight babies and maternal antepartum haemorrhage. Currently there are no recommended medical treatment options, rather women receive advice that centres on a 'wait and see' approach. For women with a history of unexplained recurrent miscarriage providing supportive care in a subsequent pregnancy improves live birthing outcomes, but the provision of supportive care to women experiencing threatened miscarriage has to date not been examined.

**Discussion:**

While it is known that 50-70% of miscarriages occur due to chromosomal abnormalities, the potential for therapeutic intervention amongst the remaining percentage of women remains unknown. Complementary and alternative medicine (CAM) therapies have the potential to provide supportive care for women presenting with threatened miscarriage. Within fertility research, acupuncture demonstrates beneficial hormonal responses with decreased miscarriage rates, raising the possibility acupuncture may promote specific beneficial effects in early pregnancy. With the lack of current medical options for women presenting with threatened miscarriage it is timely to examine the possible treatment benefits of providing CAM therapies such as acupuncture.

**Summary:**

Despite vaginal bleeding being a common complication of early pregnancy there is often reluctance from practitioners to discuss with women and medical personal how and why CAM may be beneficial. In this debate article, the physiological processes of early pregnancy together with the concept of providing supportive care and acupuncture are examined. The aim is to raise awareness and promote discussion as to the beneficial role CAM may have for women presenting with threatened miscarriage.

## Background

Vaginal bleeding is a common complication of pregnancy, with an estimated 20% of pregnant women experiencing this in the first 12 weeks of their pregnancy [1]. Threatened miscarriage involves vaginal bleeding with a viable fetus under 20 weeks gestation and a cervix that remains closed [2]. While a pregnancy can be established through ultrasound diagnostics detecting the presence of an interuturine gestational sack, it is recommended that diagnosis of threatened miscarriage viability be determined by the presence of a fetal heart beat [3]. While the risk of subsequent pregnancy loss is reduced following this confirmation of cardiac activity, these pregnancies remain at risk. Women presenting with light vaginal bleeding have twice the rate of miscarriage when compared to pregnancies with no vaginal bleeding, with this risk factor increasing to four times with heavy vaginal bleeding [4]. Women presenting with threatened miscarriage may also remain at risk for adverse pregnancy outcomes until delivery at term. These pregnancies are associated with events such as antepartum haemorrhage, premature delivery, and result in a significantly higher rate of low-birthweight babies [3,5]. Women presenting with threatened miscarriage also have twice the risk of delivering prematurely between 34 to 37 weeks [6], with risk the factors associated with birth before 37 weeks gestation including neonatal death [7].

### Clinical care for threatened miscarriage

Currently the clinical treatment for women presenting with a threatened miscarriage involves a "wait and see" approach. While a Cochrane systematic review found evidence of reduced miscarriage rates for women using progesterone supplementation (risk ratio (RR) 0.53; 95% confidence interval (CI) 0.35-0.79), the authors concluded that due to the small number of participants (421) and the poor methodological quality of eligible studies (four) there was insufficient evidence at present to support the routine use of progestogens for the treatment of threatened miscarriage. It was recommended that further randomised controlled trials were required to examine the safety of using this treatment during early pregnancy [8]. In a Cochrane review of anti coagulant therapies [9], the use of unfractionated heparin and low dose aspirin was found to be beneficial in women with antiphospholipid antibodies and recurrent pregnancy loss, however the benefits of using this therapy in women with antiphospholipid antibodies without a history of recurrent miscarriage was not thought to be sufficient to recommend its use. In this review caution was also advised for the use of prednisone for all pregnant women with adverse effects including premature delivery and gestational diabetes. Bed rest has also been examined as a treatment intervention, with a Cochrane systematic review examining two small studies involving a total number of 84 women. No statistically significant difference was found for the risk of miscarriage in the bed rest group (hospital or home), versus a no bed rest group (placebo or other treatment). (RR)1.54, 95% confidence interval (CI) 0.92 to 2.58), leading the authors to conclude that there was not enough information to recommend bed rest as a treatment option [10].

There are clinical guidelines for establishing fetal viability and treatment options for women experiencing a miscarriage but currently no clinical guidelines for treatment options that can be offered to women for threatened miscarriage [11,12]. Women who present at Early Pregnancy Assessment units or to a hospital department, GP or midwife with vaginal bleeding and are subsequently confirmed to have a viable pregnancy through ultrasound receive advice that focuses on the role of chromosomal abnormalities in miscarriage and that they can do little but "hope for the best." The present medical recommendation is to wait until a woman has experienced three concurrent miscarriages, before a diagnosis of recurrent miscarriage is made and investigations undertaken to identify possible causative factors [13]. The causative factors for pregnancy loss include: parental chromosomal anomalies, maternal thrombophiliac disorders, endocrine disorders, autoimmune conditions, and structural uterine anomalies, but for up to 50% of women a causative factor for their repeated pregnancy loss is never found [14,15]. While some clinicians may begin investigations following two miscarriages, rather than waiting for a third to occur, there remains an acceptance that miscarriage is an acceptable part of a woman's reproductive health history. In view of women delaying childbearing [16], rising maternal age associations with an increased miscarriage risk [17] and the negative psychological aspects that occur with miscarriage [18], it is relevant that potential treatment options are explored for women presenting with a threatened miscarriage. While accepting that the major causative factor for miscarriage is non recurring chromosomal abnormalities, reported as being responsible for 50-70% of all miscarriages [19] and that causative factors such as; smoking, pre-existing medical conditions, infectious agents, environmental pollutants or uterine abnormalities contribute to miscarriage rates, the reality is that the causative factor for the majority of miscarriages remains unknown [20,21]. Amongst this group of women where the cause of miscarriage is unknown there exists the potential for therapeutic intervention.

### Supportive care as an evidence based treatment option for recurrent miscarriage

Supportive care has been provided as a specific intervention for women experiencing recurrent miscarriage but not as yet for women experiencing threatened miscarriage. There are various interventions described when using 'supportive care' in studies involving pregnant women. In a Cochrane systematic review of support provided during at risk pregnancies, support was defined as any of the following; emotional (which could include counseling and reassurance), providing information or advice, as well as practical support (an example was given of transportation to clinic appointments) [22].

When supportive care was provided for women with recurrent miscarriage this care had beneficial outcomes with research demonstrating increased live births for woman when compared to a control group receiving standard antenatal care (Table 1). Of the four studies, three were controlled trials, two demonstrating a live birth rate of 86% compared to 33% for women receiving standard antenatal care, (*p *< 0.05) [23,24]. With the third reporting a 26% miscarriage rate in the intervention group compared to 51% in the control group (*p *< 0.002) [15]. The fourth study was a prospective cohort study and reported a 75% pregnancy rate beyond 24 gestational weeks [14].

**Table 1:** Supportive Care use in Recurrent Miscarriage Studies 1984 - 1999

Author	Trial design	Supportive care	Control Group	Supportive care	Outcomes
Stray-Pederson 1984	Non randomized controlled trial	N = 37	N = 24	Advice: rest, avoid heavy lifting, travelling, refrain from sexual intercourse. Bed rest for 2 weeks at the gestational age they had previously miscarried.	Reported as live births at term. Supportive care 32(86%) Routine antenatal care 8 (33%) (*p *< 0.001)

Liddell 1991	Randomized controlled trial	N = 42 (44 pregnancies)	N = 9	A weekly visit (5-13 gestational weeks). Consistent medical personal, weekly medical monitoring- (an ultrasound, serum HCG and progesterone tests), weekly stress reduction session with a physiotherapist, relaxation tape for home use, access to visit the early pregnancy clinic for reassurance.	Reported as live births at term Supportive care 38 (86%) Routine antenatal care 3 (33%) (*P *= 0.005)

Clifford 1997	Non randomized controlled trial	N = 160	N = 41	Weekly ultrasound until 12 weeks at a specialized early pregnancy clinic.	Reported as miscarriage rates 42 (26%) supportive care group 21 women (51%) routine antenatal care (*P *= 0.002).

Brigham 1999	Prospective longitudinal observational study	N = 226	N/A	Supportive care protocol that included a fortnightly ultrasound for fetal viability until 12 weeks gestation.	Reported as pregnancy rates. 75% beyond 24 weeks

These women were all presenting with a pregnancy following three or more miscarriages and had undertaken previous medical investigations where no cause had been found for their previous pregnancy losses. The support provided for these women was not specifically defined; rather terminology was used within these articles that referred to antenatal counseling, emotional support, psychological support, supportive care, tender loving care (TLC) and health advice. The specific interventions ranged from a medical investigation - that of a weekly ultrasound, to care that included advice on stress reduction, issuing women with a relaxation tape for home use, and advice about bed rest and abstaining from sexual intercourse. The specific support detailed within these research papers is outlined in Table 1.

When women with unexplained recurrent miscarriage were interviewed about the supportive care they would prefer to receive in a subsequent pregnancy [25], their responses included both medical and non medical support. Women saw medical supportive care as including; the planning of consultations and ultrasounds with their gynaecologist, ßHCG blood monitoring prior to their first ultrasound to determine fetal viability, frequent (weekly or fortnightly) ultrasounds and a preference to have only one or two gynaecologists involved in their care.

They also requested what can be seen as non medical support; advice about diet and lifestyle activities- including the recommendation of trusted websites to obtain this information, to be asked about their emotional needs and how they were doing, to be taken seriously, to feel they were being listened to and understood, to be offered counseling and relaxation tools, including a relaxation tape.

Although the studies to date report on the use of supportive care with recurrent miscarriage there is relevancy to threatened miscarriage as in one of these studies [23] 40% of the women in the supportive care group experienced symptoms of threatened miscarriage, often bleeding for several weeks. It was also reported that while an assumed miscarriage rate in the general population can be taken as 15%, after accounting for ectopic pregnancies and testing for genetic abnormalities, only 5% of the miscarriages amongst the women receiving supportive care remained unexplained. While chromosomal testing was not available for the control group and therefore a direct comparison cannot be made, the miscarriage rate for this group was 67%. The low rate of unexplained miscarriages and the increased incidence of successful live birthing outcomes for women receiving supportive care raise interesting possibilities that this care may have a role to play as a treatment in threatened miscarriage. While the mechanisms behind why supportive treatment reduces miscarriage rates remain unknown [15], the suggestion has been made from two authors, that the provision of regular medical monitoring and reassurance may reduce stress for these women which in turn positively impacts on pregnancy outcomes [23,14]. Despite these studies involving a small number of women there is acceptance from the medical community that supportive care is a valid treatment option. In a review of treatment options for unexplained recurrent miscarriage the Special Interest Group Early Pregnancy (SIGEP) of the European Society for Human Reproduction and Embryology (ESHRE) recommends supportive care as the only intervention that does not require further randomized control trials [26].

Further explanations for why providing reassurance to women may assist in establishing an optimal environment for a pregnancy may lie in examining the physiology of early pregnancy and the potential of stress to impact on a pregnancy at this time.

### Pregnancy physiology and stress responses

In early pregnancy progesterone levels are dependent on ovarian production until the placenta takes over production at 10-12 weeks [27]. While the immune responses to implantation and early embryo development are not fully understood it is thought that immune and inflammatory responses capable of destroying the developing embryo are selectively compromised by progesterone [28]. It is therefore theoretically possible that a mother's response to stress in early pregnancy has a different effect than in later pregnancy when the placenta has taken over progesterone production. A suggestion has been made that stress in early pregnancy may have the potential to promote overstimulation of the hypothalamic - pituitary - adrenal axis, resulting in decreased progesterone production, and/or altered immune responses. This may, in turn, lead to an unfavorable environment for maintaining a pregnancy [29]. It may be that for women not miscarrying due to chromosomal abnormalities supportive care can assist women to moderate stress responses, which in turn may have the potential to optimise natural hormonal production with beneficial outcomes. Due to vaginal bleeding being a common complication of early pregnancy and the lack of medical care options available, for many women there is a perceived lack of care available to them when they present with vaginal bleeding and a viable pregnancy. Given that for many women waiting until they have experienced several miscarriages before receiving supportive care is not an acceptable option, it is possible women may seek complementary therapies as a treatment option rather than accepting the current medical 'wait and see approach.'

As discussed above supportive care has been defined as providing emotional support and information. Within recurrent miscarriage research supportive care demonstrated beneficial pregnancy outcomes in early pregnancy. With women with unknown reoccurring pregnancy loss expressing an interest in receiving information on diet and lifestyle, including relaxation techniques, there is the potential for CAM practitioners to provide emotional support and diet and lifestyle advice, including relaxation techniques, as supportive care for women presenting with threatened miscarriage. In addition to providing supportive care, it is possible that specific CAM therapies such as acupuncture may have additional beneficial effects.

### The role of acupuncture in the treatment of threatened miscarriage

Acupuncture is a treatment modality that belongs to the wider scope of practice within traditional Chinese medicine (TCM), with TCM also including the use of herbal medical, massage and manipulation techniques. Acupuncture involves the insertion of needles into specific body points to stimulate an energetic response involving qi, also termed energy or the body's life force. The aim is to initiate therapeutic responses through being able to promote the smooth flow of qi within well defined pathways, known as meridians. The decision as to which acupuncture points are chosen comes from a diagnostic framework that considers the persons individual characteristics as well as presenting signs and symptoms. Historically there are texts detailing the use of acupuncture treatment date from 200 BCE, but these texts do not specifically address women health issues until the Song Dynasty, when the writings of scholars and physicians identified disorders specifically related to women's fertility, pregnancy and postpartum [30]. In particular there was a emphasis on blood production and movement within women's bodies that underpinned diagnosis and treatment, resulting in careful observation and documentation for uterine bleeding as part of menstrual cycles, during pregnancy and postpartum.

Sun Simiao (581-682 CE) was an early writer who stressed the importance of female health for successful reproduction, with TCM advice both for successfully maintaining a pregnancy and for treatment when there was vaginal bleeding in early pregnancy [31].

These historical concepts of treating women for vaginal bleeding in pregnancy, acknowledged as potential miscarriage, continued to be explored throughout the development of traditional Chinese medicine, forming the basis for acupuncture treatment protocols given in acupuncture text books published today [32-36].

Although these treatment recommendations exist within text books there is as yet no convincing evidence based literature that acupuncture is an effective treatment for threatened miscarriage. The only reported study to date (Table 2) consist of a clinical trial from within China [37]. Clinical trials from China are often developed to meet different criteria than clinical trials involving acupuncture in western medical settings. As there is often an acceptance that acupuncture is an effective treatment trials may be undertaken to demonstrate how, rather than if, they should be used within a public health system [38]. The study in Table 2 is an example of this, comparing different styles of acupuncture with no attempt made to evaluate the acupuncture treatment against a control group. The methodology of this study is poor with a high risk of bias as outlined in Table 2 giving no confidence in the reported results.

**Table 2:** A Randomised Controlled Trial of Acupuncture use in Threatened Miscarriage

Study	Method	Participants	Intervention	Outcomes	Risk of Bias
Li & Xie, 2005	Randomised controlled trial	60 women with vaginal bleeding of less than 24 hours recruited between 5 and 27 gestational weeks from hospital clinic.	Women allocated to two treatment groups with each group receiving a different style of acupuncture -once a day for ten days.	Reported as a 90% live birthing rate in one group compared to 60% (*p *< 0.01). No attempt to stratify women for confounding variables of maternal and gestation age.	High No details of Randomization process No allocation concealment No blinding of participants or outcome assessors No reporting of any incomplete data or loss to follow up.

Although quality research does not yet exist to support the use of acupuncture for threatened miscarriage, there is some limited research examining the use of acupuncture amongst women experiencing fertility related issues that may have relevance. This research examines both how women experience the care they receive and physiological responses in terms of beneficial hormonal responses.

In an attempt to gather information about how infertile women viewed the acupuncture treatment they received a randomised controlled trial examined the effectiveness of acupuncture for reducing infertility-related stress. This involved 32 women receiving acupuncture compared with a wait-list control. Women in the acupuncture group reported significant changes on a validated questionnaire assessing infertility-related stress; the Fertility Problem Inventory (FPI), demonstrating less social concern (mean difference [MD] - 3.75, 95% confidence interval [CI] - 7.58 to 0.84, *p *= 0.05), and less relationship concern (MD - 3.66, 95% CI - 6.80 to - 0.052, *p *= 0.02). In the qualitative arm of this study women described positive benefits in terms of feeling relaxed, calm, and gaining a sense of control [39].

In a small qualitative study exploring the experiences of women receiving acupuncture treatment to improve their fertility all eight women interviewed expressed positive emotional effects specifically relating to treatment. These benefits included their ability to cope with daily life stress, any medical treatment undertaken and regaining a sense of personal control and were reported even when there was no positive pregnancy outcome [40]. While limited by the small number of participants this exploratory research suggests that women see acupuncture as providing supportive care and beneficial effects in terms of their ability to cope when confronted with infertility related issues.

In an attempt to examine physiological responses to acupuncture treatment, women in a small prospective study, were tested for hormonal changes while undergoing IVF treatment. The 34 women receiving acupuncture demonstrated significant beneficial changes in serum cortisol and prolactin levels (stress hormones thought to impact on IVF pregnancy rates), compared to the 33 women receiving IVF medication alone [41]. In addition, this study found a significant reduction in miscarriage rates amongst those women receiving acupuncture treatment (*p *< 0.05). While further studies are required to confirm this it may be that acupuncture has potential to promote beneficial hormonal responses in early pregnancy that would be advantageous to women presenting with threatened miscarriage.

## Discussion

In clinical practice acupuncture can be seen as a complex treatment intervention [42]. Where treatment is individually formulated by the practitioner in response to their interpretation of a person's presentation and where treatment effects are not only derived from the use of needles, but also associated interventions such as diet and lifestyle advice. Non specific components, such as providing attention, touch, and the development of a therapeutic supportive relationship are also thought to contribute to acupuncture treatment effects. As such these effects can be seen as providing supportive care and may be practitioner specific.

It remains unclear just how possible it is to tease any specific needling effects apart from these non specific treatment effects. For example, when women receiving acupuncture from the same practitioner discussed receiving treatment, benefits were attributed both in terms of the specific practitioner relationship and specific needling effects [39].

I found her really easy to talk to in that regard, so that was good. What I found really positive was talking to [her] before the treatment; that was really great (S2).

Well it was a very warm fuzzy feeling, very relaxed, warm, and feeling like it had made a big difference (S5).

It has been argued that due to these difficulties in determining the 'active' components of CAM therapies such as acupuncture, trials to evaluate treatment effects should first start with examining safety and effectiveness. A possibility when using pragmatic randomized controlled trials. When effectiveness has been established further studies can then be undertaken involving randomised controlled trials with placebo arms to seek out active treatment components [43].

To further investigate the potential effects of acupuncture therapy on women presenting with threatened miscarriage a mixed methods study is currently being conducted in Wellington New Zealand. In acknowledgement that in the process of providing acupuncture treatment, non specific therapeutic factors contribute to treatment effects, the randomised controlled trial aspect of this study compares acupuncture treatment to an active control group receiving supportive care only. In the qualitative arm of this study thematic analysis will be used to examine the experience of receiving both supportive care and acupuncture. Further details for this study can be found on the Australian New Zealand Clinical Trials Registry (ANZCTR), registration number: ACTRN12610000850077.

There is often reluctance from CAM practitioners to discuss with women and medical personal how and when CAM therapies such as acupuncture may be beneficial for women experiencing vaginal bleeding early in pregnancy. Perhaps this is due to concerns that any treatment will be associated with a subsequent miscarriage but it is also possible that practitioners are not aware of the role of supportive care as accepted evidence based practice within recurrent miscarriage care. They may also be unfamiliar with the physiology of early pregnancy and the potential for stress responses to negatively impact on pregnancy outcomes.

Further research is required to examine any possible benefits from both the delivery of supportive care for women presenting with threatened miscarriage and providing acupuncture at this time. However for practitioners working with women to promote fertility related issues and pregnancy care it is logical that interested women presenting with threatened miscarriage are provided with the information known at present, allowing them to make informed choices around possible treatment options, rather than being forced to adapt the current medical 'wait and see approach'. It is also logical that discussions are undertaken with the medical personal involved in care of these women as to the possible benefits of CAM therapies involving supportive care and more specifically acupuncture as providing possible beneficial effects.

## Summary

Current evidence recommends that supportive care be offered to women after they have experienced three or more miscarriages. Women have requested that this supportive care include diet and lifestyle advice, including trusted internet sites to visit, relaxation tools and a therapeutic relationship. For women presenting with threatened miscarriage, it is known that 50-70% women who miscarry do so due to chromosomal abnormalities, but it is unknown what percentage of the remaining women, miscarrying for unidentified reasons, could be aided by therapeutic interventions such as supportive care and acupuncture to maintain a successful pregnancy.

The physiology of early pregnancy raises the possibility that maternal response to stress may impact negativity in terms of hormonal responses required to promote an optimal environment for a pregnancy at risk. CAM practitioners who offer women emotional support, diet and lifestyle advice, including relaxation tools, have the potential to be seen as providing supportive care as part of their practice and may be ideally placed to assist women presenting with threatened miscarriage to moderate their stress levels. In addition to offering treatment women have found to be beneficial in assisting them to moderate their stress responses acupuncture as a specific therapy may also have benefits in terms of promoting specific hormonal responses in early pregnancy. To further explore possible beneficial outcomes research in the form of a mixed methods study is currently been undertaken to examine the effects of acupuncture for women presenting with threatened miscarriage in Wellington New Zealand.

In view of the lack of treatment options women have when presenting with threatened miscarriage and the increased risk for subsequent miscarriage, PPROM, preterm delivery, low birth weight babies and antepartum haemorrhage it is relevant to seek possible therapeutic treatment solutions for this common complication of early pregnancy. It is hoped that this article will raise awareness amongst practitioners of CAM as to the medical acceptance of supportive care as a therapeutic treatment and the possible additional benefits of acupuncture treatment for women presenting with threatened miscarriage. Enabling practitioners to discuss the possible benefits of providing care within their practice both for women seeking treatment and with medical practitioners involved in their care.

## Competing interests

The authors of this article are currently undertaking a mixed methods research trial into the effects of acupuncture on women presenting with threatened miscarriage, funded as PhD research though the university of Western Sydney.

## Authors' contributions

DB, CS and HD discussed the supportive care in CAM therapies and drafted the manuscript. DB devised the tables. All authors have read and approved the final manuscript.

## Author details

PhD candidate, Centre for Complementary Medicine Research University of Western Sydney.

## Pre-publication history

The pre-publication history for this paper can be accessed here:

http://www.biomedcentral.com/1472-6882/12/20/prepub
